# Antibacterial activity of aqueous and methanol extracts of selected species used in livestock health management

**DOI:** 10.1080/13880209.2017.1287744

**Published:** 2017-02-10

**Authors:** Clarice P. Mudzengi, Amon Murwira, Musa Tivapasi, Chrispen Murungweni, Joan V. Burumu, Tinyiko Halimani

**Affiliations:** a Department of Geography and Environmental Science, University of Zimbabwe, Harare, Zimbabwe;; b Department of Research and Specialist Services, Division of Livestock Research, Grasslands Research Institute, Marondera, Zimbabwe;; c Department of Veterinary Science, University of Zimbabwe, Harare, Zimbabwe;; d Department of Animal Production and Technology, School of Agricultural Sciences and Technology, Chinhoyi University of Technology, Chinhoyi, Zimbabwe;; e Department of Livestock and Veterinary Services, Division of Veterinary Services, Harare, Zimbabwe;; f Department of Animal Science, University of Zimbabwe, Harare, Zimbabwe

**Keywords:** Ethnoveterinary medicine, minimum inhibitory concentration, *Staphylococcus aureus*; *Escherichia coli*

## Abstract

**Context:***Salvadora persica* L. (Salvadoraceae), *Colophospermum mopane* (J.Kirk ex Benth.) J. Léonard (Leguminosae) and *Dichrostachys cinerea* (L.) Wight & Arn. (Leguminosae) crude extracts are used by local farmers against many livestock infections with little or no side effects usually associated with synthetic antimicrobials. However, their efficacy has rarely been tested.

**Objective:** These plants were tested for potential antibacterial activity against clinical isolates of *Staphylococcus aureus* ATCC33862 and *Escherichia coli* ATCC25922. Minimal inhibitory concentrations (MIC) of the crude plant extracts were determined.

**Materials and methods:** Aqueous and methanol extraction of 100 g each of the bark of *C. mopane*, roots of *D. cinerea* and leaves of *S. persica* was done by placing the samples in 250 mL of either water or methanol. Nutrient broth was used as growth medium for the bacteria, and McFarland standard for bacterial standardization. 2,3,5-Triphenyltetrazoliumchloride (TTC) was the indicator salt. Each of the aqueous and methanol extracts (100 μL) was tested. Gentamycin and ampicillin were the controls.

**Results:** MIC of aqueous extracts ranged from 1.03–14.6 mg/mL against *S. aureus*, and from 12.1–34.3 mg/mL against *E. coli*. Methanol extracts ranged between 5.31 and 9.64 mg/mL against *S. aureus*, and between 7.86 and 13.6 mg/mL against *E. coli*. Aqueous and methanol extracts of *S. persica* were significantly higher (*p* < 0.05) than *C. mopane* and *D. cinerea.*

**Discussion and conclusion:***Colophospermum mopane*, *S. persica* and *D. cinerea* exhibited antibacterial activity, with methanol extracts performing better than aqueous extracts, justifying use as ethnoveterinary medicine. Further study to isolate the active components should be pursued.

## Introduction

Livestock production is an important source of livelihoods to people living in semi-arid areas of Southern Africa that are generally not suitable for crop production. These marginalized areas are usually in close proximity to national parks. In recent years, rangeland conditions have been deteriorating with new disease outbreaks on the increase due to frequent droughts and shifts in the geographical occurrence of organisms (Cumming 2005; Intergovernmental Panel on Climate Change 2007). The situation has been worsened by deliberate increases in cattle numbers by farmers who use the high cattle numbers as a hedge against losses during drought (Murungweni et al. 2012). At the Gonarezhou National Park (GNP) and Malipati Communal Area interface of the South East Lowveld of Zimbabwe (SEL), for instance, farmers respond to these grazing shortages by poach grazing their cattle in the park, and in the process also accessing traditional medicines and other natural resources. This creates a complex human-wildlife-livestock interaction. In fact, between 2000 and 2010, livestock herds were reported to go as deep as 10 to 20 km into GNP (Gandiwa et al. 2011). Besides being illegal, this pattern of livestock movement has resulted in elevated probabilities of disease transfer between cattle and wildlife (de Garine-Wichatitsky et al. 2010; Murungweni et al. 2012), and physical injuries requiring treatment. However, besides being expensive for resource poor farmers in these remote areas, orthodox drugs are not easily accessible. Therefore, the locals have always depended on ethnoveterinary medicines as alternative antimicrobials that are cheap, locally available and environment-friendly. Ethnoveterinary plants are also potent against many infections with little or no side effects associated with synthetic antimicrobials (Moreki 2012). However, there is need for scientific validation of the pharmacology of most ethnoveterinary plants. Therefore, this study seeks to test the antibacterial effect of browse species that are also used as ethnoveterinary medicine for livestock health management.

Ethnoveterinary medicines constitute a significant part of indigenous knowledge systems of livestock health management worldwide. They are cost effective, easy to administer and have no prominent resistance (Moreki 2012). Approaches to ethnoveterinary medicines have a wide range of traditional local ethno-knowledge and associated skills, techniques, practices, beliefs, taboos, cultures, practitioners and socio-economic structures pertaining to livestock husbandry (McCorkle 1986; McCorkle & Mathias-Mundy 1992). A huge number of novel drug components have been isolated from natural plant sources, and their extract applied in ethnoveterinary medicines (Obeidat et al. 2012). They have been used for the control and treatment of diseases and infections caused by some of the common bacteria of livestock that include *Escherichia coli* and *Staphylococcus aureus*. For instance, *Colophospermum mopane* (J.Kirk ex Benth.) J. Léonard (Leguminosae) is used against diarrhoea (Ribeiro et al. 2010) and *Salvadora persica* L. (Salvadoraceae) has been documented for the treatment of fevers and abortion (Fratkin 1996). Scientific validations have also proved efficacy of *Tagetes minuta* L. (Asteraceae) for tick control (Moyo & Masika 2013), *Acacia nilotica* (L.) Willd. Ex Delile (Fabaceae) (Badar et al. 2011) for helminth control, as well as the control of dermatophilosis by *Dichrostachys cinerea* (L.) Wight & Arn. (Leguminosae) (Ndhlovu & Masika 2013) among a wide array of other species. In earlier studies, phytochemical tests of leaves, stem and roots of *D. cinerea* showed the presence of alkaloids, saponins, terpenoids, flavanoids, sterols, polyphenols, proteins, tannins and carbohydrates (Aworet-Samseny et al. 2011; Vijayalakshmi et al. 2013). *Salvadora persica* has also been shown to possess terpenoids, alkaloids, tannins, glycosides, flavonoids, proteins, carbohydrates and saponins (Akhtar et al., 2012; Gupta et al. 2015). Likewise, *C. mopane* contains tannins, polyphenols, flavonoids, diterpenes and anthocyanidins (Reiter, 2002; Ferreira et al. 2003). In this regard, it is asserted that ethnoveterinary plants can overcome the universal problem of antibacterial resistance, and consequently improve livestock production for sustainable rural livelihoods.

Despite an increase in ethnobotanical surveys in Zimbabwe, phytochemical and pharmacological research of ethnoveterinary plants is still emerging. Therefore, unlike conventional drugs, appropriate extraction methods, dosages and application forms of ethnoveterinary plants are not known (Mudzengi et al. 2014). This reduces reliance in use, and appreciation of ethnoveterinary medicine. Additionally, climate change and other anthropogenic factors such as deforestation and intensification of agriculture and urbanization can lead to extinction of ethnoveterinary plants. Therefore, scientifically validating efficacy of ethnoveterinary plants can influence preservation, conservation and sustainable management of these species for utilization in livestock health management.

This study is designed to test the antibacterial effect of three selected browse species used in ethnoveterinary medicines in the south East lowveld of Zimbabwe (SEL). These are *C. mopane, S. persica* and *D. cinerea*. Aqueous and methanol extraction methods were used to isolate the active ingredient. The efficacy of these two phases was also compared. The broth microdilution method (Eloff 1998) was used to determine the minimal inhibitory concentration (MIC) of the plant extracts against *S. aureus*, a Gram-positive bacterium, and *E. coli*, a Gram-negative bacterium. This information is an important step in understanding effective extraction methods, thereby improving conservation and management of ethnoveterinary plants.

## Materials and methods

### Study area

**Figure 1.**
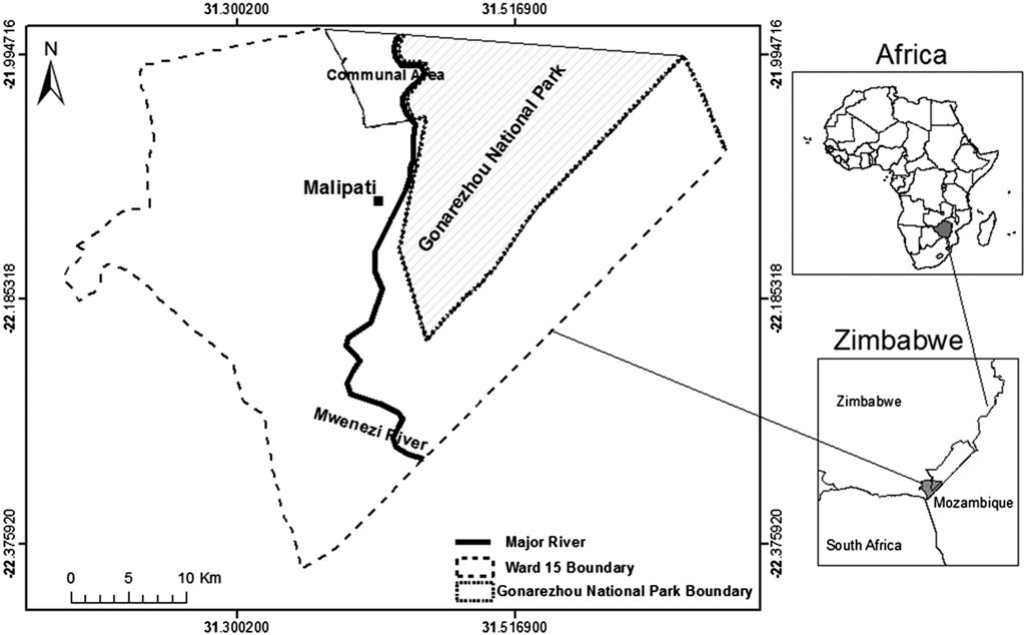
Location of the study area in the South-East Lowveld of Zimbabwe.

This study was based on Malipati communal land adjacent to Gonarezhou National Park (GNP) in the SEL (Figure 1). Malipati is located between 22°5′23.50′S and 31°22′3.16′E to the West and 22°2′57.66′S and 31°26′58.81′E to the East at an altitude of 300 m to 600 m above sea level. The area experiences mean maximum and minimum temperatures of 21.8 °C in October, and 13.3 °C in June, respectively. However, rainfall is erratic, mainly falling between November and March. It ranges from 300 to 600 mm in a season. Like the greater part of the SEL, the vegetation of Malipati is dominated by *C. mopane* and *Combretum* woodland/shrub interspaced with *Acacia* shrubs and riparian woodland (Zengeya et al. 2011). Livestock production is the main source of livelihoods in the area.

### Species identification and collection

Samples of the bark of *C. mopane*, roots of *D. cinerea* and leaves of *S. persica*, identified by the locals as having ethnoveterinary value in livestock production in the SEL were collected. They were randomly taken at different locations in May 2016 and verified at the National Herbarium in Harare, Zimbabwe where they were deposited as Specimen Voucher C Mudzengi 1 (*C. mopane*), Specimen Voucher C Mudzengi 2 (*S. persica*) and Specimen Voucher C Mudzengi 3 (*D. cinerea*). Field identification guides (Palgrave 1983; Plower & Drummond 1990; van Wyk & van Wyk 1997; Carruthers 1997) were used to identify the plant species. For each tree, location, height, canopy cover and stem circumferences were recorded. Method of collection was as advised by the locals. For each of the species, Table 1 shows the part used, the disease or condition they are used to treat and the application method.

**Table 1.** Ethnoveterinary uses of selected browse species by livestock farmers in the SEL.

Species	Disease or condition	Plant part	Application method
*D. cinerea*	Bloody diarrhoea in cattle	Root	Infusion administered orally
	Abdominal pains		
*C. mopane*	Diarrhoea in cattle	Bark	Infusion is drenched
*S. persica*	Abdominal pains	Leaves	Infusion taken orally

### Aqueous and methanol extraction of the active ingredient

The active ingredient from each sample was extracted using both water and methanol. A sharp knife was used to cut the samples into small pieces, and then put 100 g of each sample in either 250 mL of water or of methanol. Quantity of water used was minimized to reduce time of freeze drying. The samples were then put in a blender for about 10 min, and filtered using Whatman filter paper #542 which retains fine particles. The extract was stored in Eppendorf (plastic) tubes before freeze drying.

### Freeze drying

Freeze drying is a special form of drying that removes all moisture from a sample. An Edwards freeze dryer (Modulya) was used for freeze drying. Both aqueous and methanol extracts were frozen and placed in a strong vacuum. Each of the aqueous and methanol extracts (200 mL) was subjected to complete freezing at −85 °C for 4–5 h, resulting in a rigid product without denaturing of enzymes or any chemical changes. This was followed by vacuum drying at −60 °C for about 16 h to remove the solvent. Vacuum drying was done in two stages. Firstly, primary drying was carried out to remove ice by sublimation, then absorption was used to remove residual moisture during secondary drying. The lyophilized product went through final conditioning to protect it from ambient moisture, oxygen and light. The final product was stored in reagent bottles covered by para-film at room temperature. Methanol extracts dried with a sticky viscous thick semi liquid that could have been due to waxes in the plants. Limitations in the methods used were that, liquid nitrogen was not affordable, yet it would have been the most preferred method for complete freezing. Additionally, instead of using the recommended oven drying temperatures of 80 °C for water and 60–70 °C for methanol, temperatures of 50 °C were used because the temperature at which the samples remain stable were not known.

### Bacterial samples

Two isolates used in the study were *S. aureus* ATCC33862, a Gram-positive organism and *E. coli* ATCC25922, a Gram-negative organism. Nutrient broth was used as the growth medium for the bacteria, and McFarland standard for bacterial standardization.

### Preparation of the extract solution

99.8% of dimethylsulphoxide (DMSO) solution was dissolved in 0.9% NaCl to give 4% DMSO. Each plant extract (1 g) was dissolved in 24 mL of the 4% DMSO to make 40 mg/mL solutions.

### Antibiotic and bacterial suspension preparation

Gentamicin and ampicillin were used as the controls. 0.5 g of ampicillin was dissolved in 12 mL of 0.9% NaCl to make a 4% (40 mg/mL) suspension. Gentamicin procured as 80 mg/2 mL from a local pharmacy was diluted to 40 mg/mL. An inoculation loop was used to scrap off a loopful of bacteria from the agar surface. This was thoroughly mixed with normal saline solution in previously autoclaved bijou bottles to give a suspension whose turbidity was compared to that of the freshly prepared McFarland standard. The turbidity of the bacterial suspension was adjusted to be similar to that of the standard, corresponding to 1 × 10^5^ colony forming units of bacteria per millilitre.

### Antibacterial activity assay – determination of the minimum inhibitory concentration (MIC)

**Figure 2.**
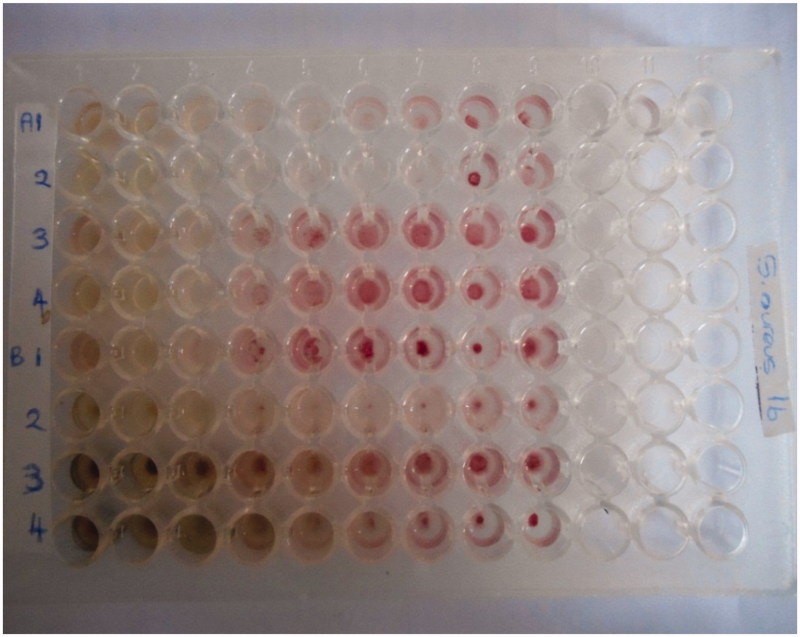
Inoculated microwell plate containing plant extract after incubation and addition of TTC.

The MIC of each of the samples was determined using the broth microdilution method (Eloff 1998). The MIC is the lowest concentration of an antibacterial agent that appears to inhibit growth of the bacteria. 100 μL of each of the aqueous and methanol plant extracts initially dissolved in 4% DMSO to make 40 mg/mL was added into the first well, and a twofold dilution was performed by transferring 50 μL of the suspension to the subsequent wells up to the 9th well. The final 50 μL of the suspension was discarded. In the 10th and 11th wells (growth controls) nutrient broth was added to 50 μL of 4% DMSO and 50 μL of 2% methanol, respectively. 100 μL of nutrient broth added into the 12th well-acted as the sterility control. Each bacterial suspension (50 μL) in 0.9% NaCl was added to the wells of a sterile 96-well microtitre plate already containing 50 μL of the twofold serially diluted plant extract in nutrient broth. Before standardization, the bacteria were incubated at 37 °C for 30 min to reactivate them. The final volume in each well was thus 100 μL. The microplates were then incubated at 37 °C for 24 h. To indicate respiratory activity (bacterial growth), we dissolved 2,3,5-triphenyltetrazoliumchloride (TTC) in water to give a 20 mg/mL solution that was added into each of the wells and incubated at 37 °C for 30 min in the dark. TTC is a salt that can be metabolized by surviving bacteria resulting in a colour change from clear to pink (Figure 2).

### Data analysis

Visual analysis of the microtitre plate wells was used to record the MIC, whereby the lowest concentration of the plant extract that inhibited bacterial growth after addition of TTC and 24 h of incubation would not change its colour to pink. A paired *t* test was used to compare potency of the aqueous and methanol phases for each of the plants against both *S. aureus* and *E. coli* using. IBM IBM Corp. (2012) SPSS was used for one way analyses of variance (ANOVA) of the MIC values obtained.

## Results

**Figure 3.**
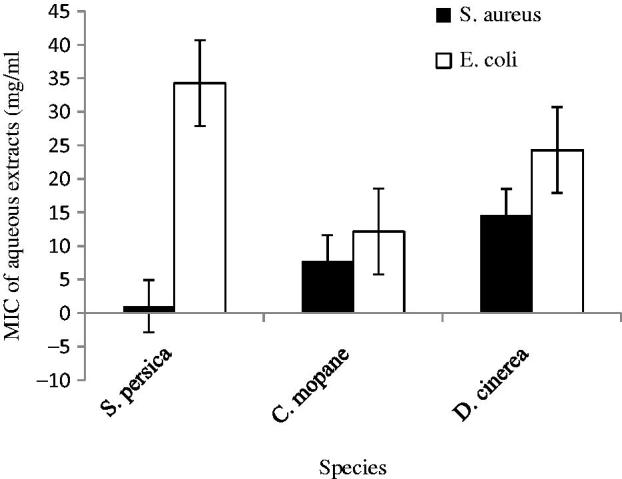
Minimal inhibitory concentrations of aqueous extracts of selected browse species against *S. aureus* and *E. coli*.

**Figure 4.**
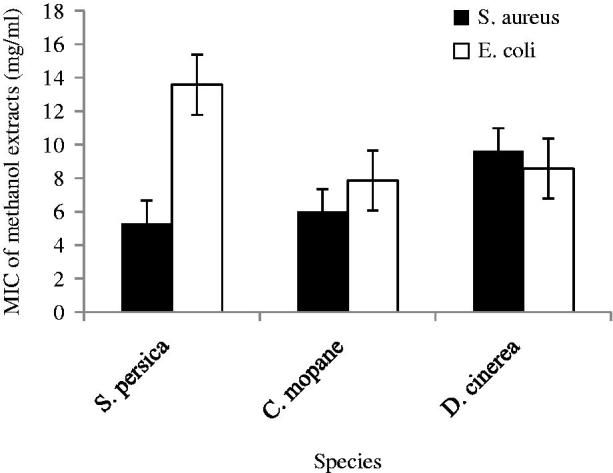
Minimal inhibitory concentrations of methanol extracts of selected browse species against *S. aureus* and *E. coli*.

All plant extracts exhibited antibacterial activity against both *S. aureus* and *E. coli.* They were generally more potent against *S. aureus* than *E. coli* (Figures 3 and 4). *Staphylococcus aureus* and *E. coli* were also sensitive to the two controls (gentamicin and ampicillin) up to one in eight dilutions. Both controls were more effective than the plant extracts. In the aqueous phase, the plant extracts showed antibacterial activity with MIC values in the ranges of 1.03–14.6 mg/mL against *S. aureus*, and 12.1–34.3 mg/mL against *E. coli*. Minimal inhibitory concentration values of the antibacterial activity of the methanol extracts of the plants ranged between 5.31 and 9.64 mg/mL against *S. aureus*, while against *E. coli* they ranged between 7.86 and 13. 6 mg/mL.

The methanol phases of *D. cinerea* and *C. mopane* had higher antibacterial activity against *S. aureus* than the aqueous phase (Figures 3 and 4). Only *S. persica* had higher potency in the aqueous than the methanol phase against *S. aureus*. There were significant differences (*t* < 0.05) between the two phases against *S. aureus* for *S. persica* (*t* = 0.000) and *D. cinerea* (*t* = 0.001). *Colophospermum mopane* had no significant difference (*t* > 0.05) between the aqueous and methanol phases (*t* = 0.056) against *S. aureus*. Methanol extraction of the plants also had higher antibacterial activity than the aqueous phase against *E. coli*. There were significant differences (*t* < 0.05) between the aqueous and methanol phases against *E. coli* for *S. persica* (*t* = 0.000) and *D. cinerea* (*t* = 0.018). However, *C. mopane* showed no significant difference (*t* > 0.05) between the aqueous and methanol phases (*t* = 0.09). Its antibacterial activity was also higher in the methanol phase.

In the aqueous phase, *S. persica* had the highest antibacterial activity against *S. aureus* at 1.02 mg/mL (Figure 3). There were significant differences (*p* < 0.05) between *S. persica* and *C. mopane* (*p* = 0.000). *Colophospermum mopane* was second at 7.71 mg/mL. Significant differences (*p* < 0.05) were also observed between *S. persica* and *D. cinerea* (*p* = 0.000) and between *C. mopane* and *D. cinerea* (*p* = 0.000). In the methanol phase (Figure 4), *S. persica* also had the highest antibacterial activity against *S. aureus* at 5.31 mg/mL, followed by *C. mopane* and lastly *D. cinerea* at 5.99 mg/mL and 9.64 mg/mL, respectively. There were no significant differences (*p* > 0.05) between *S. persica* and *C. mopane* (*p* = 0.628). However, there were significant differences (*p* < 0.05) between *S. persica* and *D. cinerea* (*p* = 0.003), and between *C. mopane* and *D. cinerea* (*p* = 0.000).

In the aqueous phase against *E. coli*, *C. mopane* had the highest antibacterial activity at 12.1 mg/mL (Figure 3). There were no significant differences (*p* > 0.05) between *C. mopane* and *D. cinerea* (*p* = 0.09), with *D. cinerea* having the second antibacterial activity at 24. 3 mg/mL. *Salvadora persica* was last at 34.3 mg/mL. It differed significantly (*p* < 0.05) from the other two species. The methanol extract of *C. mopane* also had the highest antibacterial activity against *E. coli* at 7.86 mg/mL, followed by *D. cinerea* at 8.57 mg/mL and lastly *S. persica* at 13.6 mg/mL (Figure 4). There were no significant differences (*p* > 0.05) among the methanol extracts of the three species against *E. coli*.

## Discussion

Results obtained indicate that *S. persica, C. mopane* and *D. cinerea* exhibited antibacterial activity against both *S. aureus* and *E. coli*. Most farmers in the study area use these browse species for treatment and control of livestock diseases. These results therefore justify the ethnoveterinary use of the species. Elsewhere *C. mopane* has been used in the treatment of diarrhoea (Ribeiro et al. 2010; Makhado et al. 2012), *D. cinerea* in control of dermatophilosis and diarrhoea (Ndlovu & Masika 2013) and *S. persica* for fevers and abortion (Fratkin 1996). Higher potency of the plant extracts against *S. aureus* than *E. coli* in the present study is expected. Previous studies (Parekh et al. 2005; Lino & Deogracious 2006) also reported higher efficacy of plant extracts against Gram-positive microorganisms than Gram-negative microorganisms. This may be due to the presence of a cell wall outer membrane in Gram-negative bacteria whose lipopolysaccharide covering acts as a barrier to many substances including antibiotics (Klancnik et al. 2010; Tortora et al. 2010). These plants can therefore be effective against most common pathogenic infections of livestock. Ethnoveterinary plants are thus cost effective, locally available and environment friendly alternatives to orthodox veterinary medicines.

Methanol extracts of *D. cinerea* and *C. mopane* in this study performed better than the aqueous extracts. These findings are not surprising, given that most antimicrobial active components in plant matter are saturated organic molecules, which are non-polar. Additionally, despite water being a more polar solvent than methanol, the phytochemical profile of an extract determines polarity of the compounds being extracted in a given solvent. Hence, for active lipophilic constituents that do not extract into a water extract, methanol extraction would provide more consistent antimicrobial activity compared to those extracted in water. Consistent with findings in this study, Muthaura et al. (2015), Parekh et al. (2005) and Tona et al. (1999) also indicate that although aqueous extraction might be popular, it is not necessarily the most effective method. However, higher potency of the aqueous extracts of *S. persica* against *S. aureus* would be advantageous to the locals since water is readily available and easy to handle. It is the ease of handling and use that makes water the most used solvent for bioactive compounds in traditional remedy preparations (Shale et al. 1999). However, although aqueous extracts of *D. cinerea* and *C. mopane* had lower potency than the methanol extracts, their antibacterial activity was still higher than the normal 40 mg/mL used in manufacture of commercial pharmaceuticals. Therefore, we deduce that these results could increase emphasis on studies of more effective extraction methods, thereby improving conservation and management of ethnoveterinary plants.

Minimum inhibitory concentrations of *C. mopane* and *D. cinerea* in the present study were lower than in other studies. Shai et al. (2013) recorded higher average MIC values (1.1 and 0.7 mg/mL against Gram-negative and positive bacteria, respectively) from acetone extracts of leaves of *C. mopane.* Similarly, tannins from ethanolic extraction of the root of *D. cinerea* produced higher bacteria toxicity activity with MIC values of 5.5 mg/mL against *S. aureus* and 5 mg/mL against *E. coli* (Banso & Adeyemo 2007) in comparison to methanol extraction in the present study. Efficacy of plants can be affected by many factors. For instance, differences in combinations of secondary metabolites such as phenolic compounds, tannins, alkaloids and steroids in different plant species determine the uniqueness of their phytochemistry. These metabolites are also deposited in varying proportions in different parts of an individual plant. Hence, higher concentrations would be expected in leaves than the bark, as leaves are responsible for phytochemical production. Differences in solvents used for extraction as well as geographical location could also have contributed to observed variations. Heterogeneity in the composition of compounds in the plant extracts can also lower their antibacterial activity, resulting in the plant extracts possessing little of the active ingredient. This could explain lower activity of the plant extracts compared to the controls gentamicin and ampicillin. Additionally, active compounds might exhibit higher activity in their pure form. Likewise, unlike conventional pharmaceutical products which are usually prepared from synthetic materials by means of reproducible manufacturing techniques and procedures, traditional medicinal products are prepared from materials of plant origin, which may be subjected to contamination and deterioration (Lino & Deogracious 2006). This information is important for the research and development of the most appropriate methods of extraction of active ingredients in plant species.

Results from the broth microdilution method (Eloff 1998) in this study confirmed antibacterial activity of methanol and aqueous extracts of the bark, roots and leaves of *C. mopane*, *D. cinerea* and *S. persica,* respectively. However, this study only concentrated on those plant parts commonly used by the locals in the study area. For instance, only the bark of *C. mopane* was evaluated, whereas, as Ribeiro et al. (2010) reported, its leaves, roots and stems are also used for the treatment of diarrhoea and other stomach upsets. Therefore, there is need for further studies on other parts of the same plant species before conclusions can be drawn on their pharmacological and therapeutic potential. We also acknowledge that other factors such as geographical location, time of sample collection, harvest method and storage conditions could affect efficacy of plant extracts. The active ingredient could also be extracted better in solvents other than water and methanol. Furthermore, there is need for isolation and characterization of the active phytochemical components of these plant species using more efficient bioautographic methods. Toxicity studies can also be conducted to determine their levels of safety. Moreover, extracts which are inactive *in vitro* may have properties similar to pro-drugs which are administered in an inactive form; hence their metabolites could be active *in vivo* (Lino & Deogracious 2006). We therefore recommend this advanced work as essential in order to fully validate the antibacterial efficacy of ethnoveterinary plants.

## Conclusions

Based on the MIC values obtained in this study, it is concluded that the browse species *C. mopane*, *S. persica* and *D. cinerea* have antibacterial activity against *S. aureus* and *E. coli*. The plant extracts were more potent against *S. aureus* than *E. coli.* The methanol phase of *D. cinerea* and *C. mopane* had higher antibacterial activity against *S. aureus* than the aqueous phase. Methanol extraction of all three species also had higher antibacterial activity than the aqueous phase against *E. coli*. These species can thus be used to address livestock health problems for sustainable rural livelihoods.
